# An In-Depth Study of Ring Oscillator Reliability under Accelerated Degradation and Annealing to Unveil Integrated Circuit Usage

**DOI:** 10.3390/mi15060769

**Published:** 2024-06-08

**Authors:** Javier Diaz-Fortuny, Pablo Saraza-Canflanca, Erik Bury, Robin Degraeve, Ben Kaczer

**Affiliations:** Interuniversity Microelectronics Centre, Kapeldreef 75, 3001 Leuven, Belgium; pablo.sarazacanflanca@imec.be (P.S.-C.); erik.bury@imec.be (E.B.); robin.degraeve@imec.be (R.D.); ben.kaczer@imec.be (B.K.)

**Keywords:** bias temperature instabilities (BTIs), hot carrier degradation (HCD), aging, annealing, integrated circuit, array, ring oscillator

## Abstract

The reliability and durability of integrated circuits (ICs), present in almost every electronic system, from consumer electronics to the automotive or aerospace industries, have been and will continue to be critical concerns for IC chip makers, especially in scaled nanometer technologies. In this context, ICs are expected to deliver optimal performance and reliability throughout their projected lifetime. However, real-time reliability assessment and remaining lifetime projections during in-field IC operation remain unknown due to the absence of trustworthy on-chip reliability monitors. The integration of such on-chip monitors has recently gained significant importance because they can provide real-time IC reliability extraction by exploiting the fundamental physics of two of the major reliability degradation phenomena: bias temperature instability (BTI) and hot carrier degradation (HCD). In this work, we present an extensive study of ring oscillator (RO)-based degradation and annealing monitors designed on our latest 28 nm versatile array chip. This test vehicle, along with a dedicated test setup, enabled the reliable statistical characterization of BTI- and HCD-stressed as well as annealed RO monitor circuits. The versatility of the test vehicle presented in this work permits the execution of accelerated degradation tests together with annealing experiments conducted on RO-based reliability monitor circuits. From these experiments, we have constructed precise annealing maps that provide detailed insights into the annealing behavior of our monitors as a function of temperature and time, ultimately revealing the usage history of the IC.

## 1. Introduction

High-end IC markets, such as data centers or the automotive industry, have a growing demand for reliable chips to exploit high-performance computing (HPC) applications and withstand power efficiency, chip longevity, and long-term performance [1,2,3,4], as well as reduce the carbon footprint of CMOS technologies [5,6,7]. However, the rapid downscaling of CMOS technologies, where supply voltages do not scale linearly with device dimensions, results in a concerning increase of IC degradation due to high electric fields that progressively, but inevitably, worsen IC projected reliability and lifetime [8,9]. In this scenario, IC degradation can lead circuits to progressive performance degradation or even critical failure caused by two of the major transistor aging mechanisms: bias temperature instability (BTI) [10,11,12] and hot carrier degradation (HCD) [13,14,15,16].

Various sectors, including HPC computing or the telecommunications industry, employ, for instance, Central Processing Unit (CPU) overclock techniques to boost computation performance and drastically increase chip throughput [17,18,19,20]. This is typically achieved by operating the ICs at a higher-than-nominal operation voltage. However, operating chips beyond their nominal specifications accelerates chip wear-out, reducing their performance over time and thus negatively affecting their expected lifetime [21]. To tackle this problem and assess chip degradation, chip designers apply a variety of techniques, like using conservative design margins or including path redundancy and, occasionally, on-chip time-delay monitors connected to fixed critical paths to correlate time-delay to circuit degradation [22,23,24]. However, selecting a permanent critical path is considered a deprecated technique in today’s dynamic mission profile chips, where the IC load and temperature profile could vary between system software updates that might change the IC’s mission profile [25]. Also, these techniques could increase design complexity and thus chip cost, while long-term performance remains difficult to predict in critical mission applications [26,27].

Some silicon degradation detection methodologies have been already presented in the literature. One example is the silicon odometer technology [28], which employs two identically designed ROs, one acting as a non-stressed reference and another that will be subject to overvoltage stress that will progressively degrade. Later, the reference RO will be used as a time base to extract the beat frequency and determine the accumulated degradation of the stressed RO. More recent works are focused on the detection of recycled circuits by characterizing the BTI degradation of the Input/Output (IO) transistors of the IC pads [29]. Then, to determine if the IC has been already used or if, on the contrary, the IC is brand new, the methodology aims to obtain BTI degradation without the need for including an on-chip monitor. Nevertheless, what all these solutions have in common is that they solely exploit the BTI degradation phenomenon, which can be annealed with temperature, or they need an on-chip reference to retrieve degradation metrics, which can be easily attacked to intentionally degrade the reference, so the extracted shifts are negligible [30]. Consequently, there is a growing interest in the industry in including on-chip reliability monitors without including vulnerable on-chip references coupled with anti-tampering capabilities for in-field wear-out monitoring to proactively implement predictive maintenance strategies and anticipate critical failures [7,28,31,32].

To address this emerging demand in the IC industry, we have proposed an on-chip degradation monitor technology that can reliably track the CMOS process variability of each IC during wafer sorting and monitor IC degradation in real time during in-field operations. Moreover, our technology includes a novel tamper-aware capability that prevents fraudulent IC temperature annealing from being untraceable [30,33]. Our solution provides a combined readout of BTI- and HCD-degraded monitors that exploits the orthogonal degradation kinetics of these two aging phenomena to expose the chip’s stress history and determine its remaining lifetime. Nevertheless, despite the efforts to increase orthogonality between HCD and BTI degradation by means of regular ring oscillator (RO)-based monitors, such as conventional inverter chains, where BTI aging predominates over HCD, further refinement of the degradation monitor design is necessary. This refinement aims to increase wear-out due to HCD and achieve significantly orthogonal degradation kinetics across different monitors [34,35,36,37,38,39].

In this paper, we present (i) our latest IC test vehicle, produced using a commercially available 28 nm High-k Metal Gate (HKMG) technology, which harbors hundreds of regular and tailored RO-based monitors utilized to statistically test circuit degradation dominated by BTI and HCD. The array includes regular NAND gate-controlled ROs and our newest modified RO-based monitor called ‘True-HCD’, which is specifically designed to degrade only through the HCD phenomenon. These two monitors together comprise our non-intrusive on-chip degradation monitoring technology, where the understood degradation kinetics allow us to determine the degradation of the IC and detect fraudulent tampering. Moreover, we review (ii) an on-chip heater structure designed in the Front End of Line (FEOL) with N+ diffusion resistors that allows for the execution of fast and reliable on-chip temperature annealing experiments employed to characterize the annealing behavior of our technology [40]. Finally, we present an in-depth study of (iii) RO-based degradation and annealing behavior, comparing BTI-dominated regular-RO-based monitors and our True-HCD monitors, where the acquired annealing knowledge can be utilized during in-field applications to enhance the accuracy of the IC age and clearly detect fraudulent annealing of the IC.

The paper is organized as follows: Section 2 describes the experimental setup, including our latest IC array design comprising regular and True-HCD aging monitors, and the built-in heating system for on-chip temperature annealing. Section 3 reviews our on-chip degradation monitor with tamper-aware capability technology, and Section 4 details the results gathered from the degradation and annealing tests conducted on regular-RO-based and True-RO monitors.

## 2. Experimental Setup

**Figure 1:**
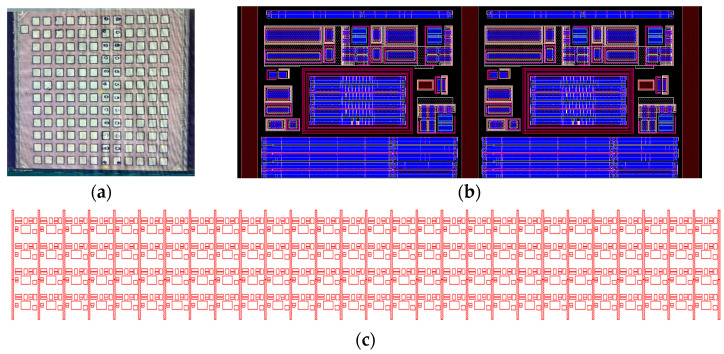
(**a**) Micrograph of the fabricated chip of our last-generation RO-array IC. The fabricated chip consists of six different modules, each with 24 pads for probe card testing. (**b**) Layout illustration of two adjacent unit cells with digital and analog circuitry surrounding a single RO in the middle, while the cells are located in between on-chip heater stripes. (**c**) GDS illustration of a RO-array module that harbors 120 individual RO unit cells, each containing a RO monitor, and reveals the vertical on-chip heater stripes connected in parallel devoted to conduct on-chip annealing.

All experimental tests performed in this study were conducted utilizing our latest versatile RO-array circuit, fabricated on a 1 mm^2^ chip. The IC allows for digital access to hundreds of regular and tailored True-HCD aging monitors dedicated to accumulating circuit degradation and detecting temperature annealing events, as shown in Figure 1a.

### 2.1. Chip Design and Architecture

To efficiently use the 1 mm^2^ area, the chip design incorporates a total of 6 individual submodules, each comprising 24 pads for both analog and digital operations. Consequently, the full die accumulates a total of 144 pads, each pad measuring 55 µm × 55 µm, including more than 600 degradation monitors [30,40] (see Figure 1a). Each monitor is integrated into a block referred to as a ‘unit cell’, which contains the necessary digital and analog circuitry to operate each RO individually under different bias conditions.

The layout of an RO-array submodule includes replicated unit cells interconnected in a mosaic pattern of 4 rows and 28 columns, totaling 112 unit cells per submodule. Unit cell selection is facilitated by means of a row and column selection circuitry constructed by two-layer shift registers. These shift registers permit individual or multiple unit cell activation for operation controlled by a 32-bit selection word, i.e., 4-bit for row and 28-bit for column selection, allowing for single or parallel testing, as demonstrated in [41,42,43]. Furthermore, the connection of the unit cell ROs to the biasing paths is achieved through three operation modes, i.e., stress, measurement or standby, which establish a physical connection between the ROs’ VDD and the biasing paths via IO transmission gates.

The array is also equipped with two individual on-chip Force-and-Sense (F&S) biasing paths designed to mitigate on-chip voltage drop, which are connected to all unit cells of the array for individual stress and measurement biasing. Thanks to the capability to select single or multiple unit cells, different unit cells can be selected for similar or different operations that can occur simultaneously, like the two adjacent unit cells shown in Figure 1b. For instance, when a unit cell is selected for stress operation, the RO is connected to the stress paths, and, at the same time, another unit cell can be selected for measurement where that RO is connected to the measure path. Also, parallel stress tests on several ROs can be conducted simultaneously to significantly reduce the total aging test time.

### 2.2. Unit Cell Building Blocks

**Figure 2:**
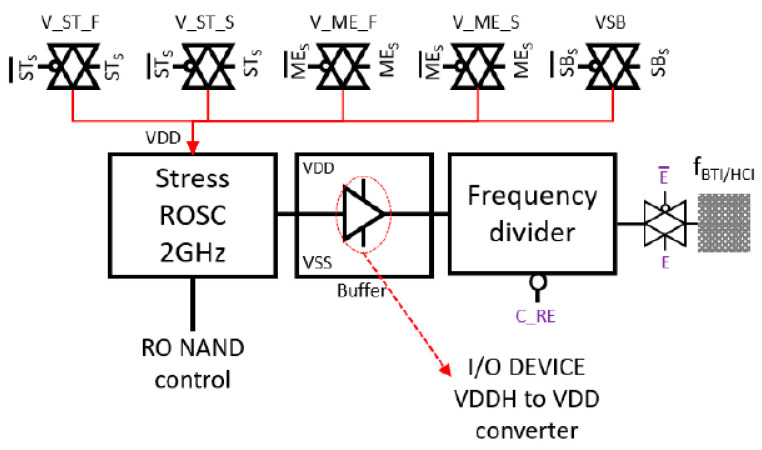
Simplified block representation of a regular-RO unit cell that includes a 51-stage RO with NAND feedback loop control. RO voltage can be provided via three sets of transmission gates: stress F&S, measurement F&S, and standby. In addition, the unit cell includes a VDDH-to-VDD voltage converter that prevents the frequency divider from degrading during high voltage RO stress.

In our array design, the unit cell circuitry can operate different RO monitor types. On the one hand, Figure 1b shows the layout of two adjacent unit cells that each contain a regular-RO-based degradation monitor. It consists of a 51-stage RO with a fundamental oscillation frequency of ~1.8 GHz. The feedback loop of the RO is controlled by a NAND gate that permits enabling or disabling RO oscillation, thus allowing for dynamic (closed-loop) or static (open-loop) reliability tests. Moreover, each RO is then connected to a 15-stage frequency divider, i.e., 2^15^ = 32,768 of maximum frequency division, which allows users to select the optimum output frequency for reliable off-chip characterization. Each unit cell has also been equipped with full thick oxide transmission gates that permit establishing the desired biasing to the target RO, as depicted in Figure 2. Moreover, a forward-biased diode has been included in the unit cell for accurate temperature sensing.

**Figure 3:**
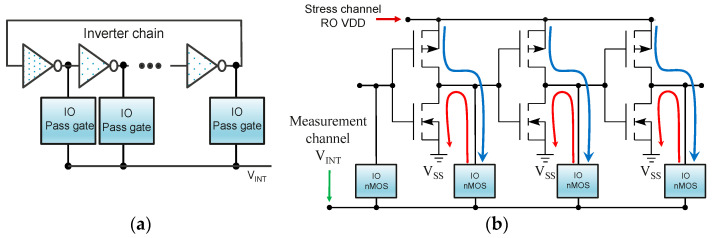
(**a**) Schematic representation of the True-HCD monitor, including thick oxide pass gates in between RO stages to force V_INT_ between each RO stage. (**b**) Current flow in blue and red exhibiting the current path when stressing all pMOS or all nMOS.

The F&S biasing system consists of four on-chip paths that establish a connection from the top pads to the corresponding pass gates of each unit cell. As depicted in Figure 2, the stress and measurement paths have their own F&S full transmission gates that allow the external biasing instrumentation to sense the RO VDD and compensate for voltage drop due to the BEOL, pass gates, and cable accumulated resistance. For large RO degradation, the stress force pass gate, i.e., V_ST_F in Figure 2, has been properly sized to sustain RO biasing voltages up to 3 V and DC currents up to 3 mA. On the other hand, Figure 3a shows the schematic design of our tailored True-HCD aging monitor. This design permits stressing the building transistors of the 23-stage INV-based RO with DC HCD. Moreover, we have included thick oxide nFET switches that permit external biasing of the intermediate nodes of the RO with V_INT_ voltage (see Figure 3b) [33,44]. All the I/O pass gates are enabled/disabled simultaneously, so V_INT_ can be biased to execute DC HCD to all the nMOS or to all the pMOS building transistors of all inverter stages simultaneously, as described in Table 1. As shown in Figure 3b, to perform DC HCD stress, the two test channels of the unit cell have been utilized: RO VDD is provided via the stress channel, while the V_INT_ voltage is supplied via the measurement channel. If the pMOS devices are stressed (blue current path in Figure 3b), a DC current flows through the pMOS from RO VDD to V_INT_; on the contrary, if the nMOS devices are stressed (red current path in Figure 3b), a DC current flows from V_INT_ to VSS through the nMOS devices. The True-HCD aging monitor has proven to be a unique RO-based aging monitor stressed mainly by HCD, with unique degradation kinetics, different from those of BTI-driven monitors.

### 2.3. On-Chip Heaters for Accelerated Degradation/Annealing Experiments

Conventional accelerated degradation and/or annealing of circuit tests are conducted at the wafer level using automatic or semiautomatic wafer probers equipped with thermochucks, which allow for the establishment of a variety of temperatures. Usually, thermochuck temperatures can be defined up to 200 °C, or, in some modern systems, up to 300 °C, but with a significantly slow rise temperature slope of ~0.1 °C/s and long temperature stabilization times, i.e., soaking time, of ~hours. These slow temperature setting times jeopardize the execution of accurate and fast reliability tests, such as device or circuit aging/annealing, because they prevent switching between different temperatures fast enough to meet the time-tight requirements of reliability testing [45,46,47,48]. Several approaches have been recently presented to overcome the above-mentioned temperature limitations of thermochucks by implementing on-chip heaters [49,50,51,52,53,54] for aging/annealing variability testing. Nevertheless, those approaches are very specific to the characteristics of single devices [52,53,54], or they need an extra fabrication process to co-integrate the heater stacked with the fabricated silicon, which may make heat transfer to the target device and the even distribution of heat across the chip difficult [51].

**Figure 4:**
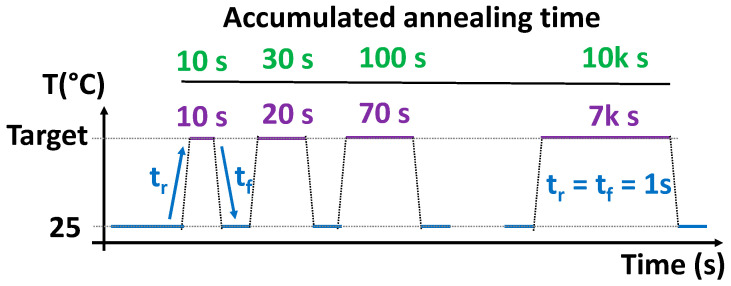
High-temperature annealing cycles with exponentially increasing times are applied to our RO-based aging monitors by means of our on-chip heater structure. During the green periods, RO monitor frequency is characterized at room temperature, while in the red plateaus, the annealing temperature is raised to, e.g., 325 °C, while the array is kept unbiased.

In our approach, the full-custom on-chip heater structure consists of 29 N+ diffusion resistors, which are integrated among the unit cells as illustrated in the layout of Figure 1c as vertical red strips. Each heater strip has a width of 2.5 µm and a length of 110 µm, with a resistance of ~800 Ω. The 29 heater strips are connected in parallel, resulting in a total resistance of ~40 Ω, when the parasitic resistance of contacts and metals is considered. Furthermore, the heater is electrically isolated from the array, which permits it to operate independently of the array, allowing the execution of biased/unbiased circuit temperature tests. Moreover, the heater structure allows for on-chip homogeneous and fast temperature setting of ∆T = +300 °C with setting times of approximately 1 s, a significantly faster temperature setting system when compared with commercial thermochucks or oven annealing [55,56]. The versatility of our FEOL heater allows us to execute logarithmic temperature tests where the annealing temperature is switched from 25 °C to, for instance, 325 °C for annealing experiments on our regular- and True-HCD aging monitors [40,55], as depicted in Figure 4.

**Figure 5:**
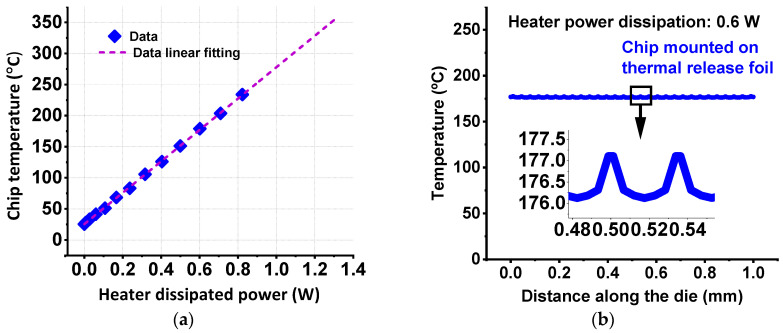
(**a**) Linear dependence of on-chip heater temperature as a function of dissipated power. (**b**) Temperature variations due to the location of the on-chip heater’s vertical stripes along the *X*-axis of the die extracted from FEM simulation revealing only 1 °C of temperature variation between two heater stripes located in the middle of the chip, as revealed by the zoom-in inset in (**b**).

We also conducted a thorough calibration of the heater in two phases: first, the I-Vs of the unit cell diodes were obtained for temperatures ranging from 25 °C to 175 °C using a FormFactor PA300 semi-automatic prober (SUSS MicroTec, Garching, Germany) with a controllable temperature. Then, the forward junction voltage (Vj) of the tested diodes was obtained, showing a linear dependence on the chuck’s temperature, resulting in a voltage–temperature coefficient of −1.38 mV/°C. Second, the heater power dissipation was measured for an input sweep voltage up to 6 V, while the Vj of diodes was constantly monitored and used to convert heater power into local temperature. Figure 5a shows the linear dependence between the converted temperature and the dissipated heater power.

To investigate and prove the accuracy of the fabricated heater, a 3D finite element model (FEM) representing the on-chip heater structure was created using a commercial FEM software, MSC Marc^®^. The thermal model was used to investigate the temperature uniformity along the die and the impact of temperature variations since the heater stripes are placed 35 mm away from each other. In this regard, Figure 5b reveals that the maximum temperature at, for instance, 0.6 W is 177 °C and the temperature variation among the heater strips do not exceed 1 °C [40].

## 3. The On-Chip Degradation Monitor with Tamper-Aware Capability Concept

**Figure 6:**
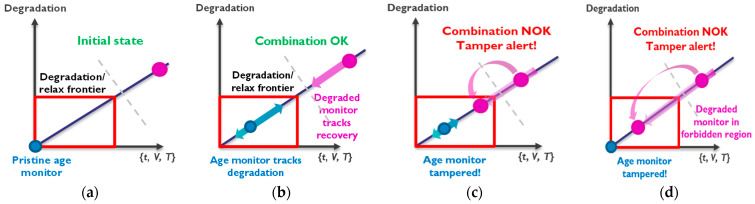
The on-chip degradation monitor concept with tamper detection capability is described in four different scenarios. (**a**) Initial: fresh (blue) + pre-stressed (pink) monitors. (**b**) After chip use: expected chip wear-out, pre-stressed monitor outside the illegitimate combination zone (red rectangle). (**c**) Illegitimate combination after tampering attempt: pre-stressed monitor enters illegal combination zone due to annealing. (**d**) Strong tampering detected by pre-stressed monitor.

In this section, we review our on-chip degradation monitor technology, which is able to obtain the degradation status of an IC and report the ‘time since fabrication’. Moreover, the on-chip degradation technology also includes a novel tamper flag, which could indicate a fraudulent IC anneal with a high temperature, e.g., due to desoldering/soldering cycles. For this purpose, two or more sets of RO-based monitor circuits are deployed: sets of pristine and heavily pre-stressed monitors, as represented in Figure 6 with blue and red ovals [30,33]. To determine the wear-out of the IC as well as to feature the tamper flag capability, our RO-based monitor technology exploits BTI and HCD [57,58,59,60,61] degradation phenomena at the circuit level. When the monitors are operated in static mode, i.e., with an open feedback loop where there is no oscillation, the main degradation mechanism is BTI for regular-RO monitors or with DC HCD degradation in our new True-HCD monitor. In contrast, when the monitors accumulate degradation in dynamic mode, i.e., the feedback loop of the monitor is closed, the RO-based monitor is oscillating, and both BTI and HCD degradation occur. In this scenario, our technology accounts for IC degradation using, for instance, two initially non-degraded monitors that will progressively degrade with time during circuit operation: one due to BTI and one due to HCD. The tamper flag capability consists of a set of heavily pre-stressed monitors, one highly degraded only by BTI and another also degraded by HCD. Then the system will track their respective relaxation behaviors during in-field operation of the IC.

As depicted in Figure 6a, the initial conditions of the two deployed sets of monitors are: the pristine monitors, which are unstressed at time zero, i.e., the blue oval, and the heavily pre-stressed set of monitors, i.e., the pink oval, which will be used to account for the relaxation of the monitors. This later set of monitors will be pre-stressed during wafer test, and they will accumulate a considerable amount of known initial degradation. By evaluating the on-chip degradation and relaxation of the two sets of monitors, we can assess whether the IC has undergone the expected degradation and relaxation based on understood physics, if it has relaxed excessively, indicating annealing, or if it has degraded too much, revealing prolonged use. Thus, three different scenarios can occur [30]:

- After nominal and legitimate IC use: following regular IC operation within the technology-defined voltage and temperature margins, the degradation monitors will progressively accumulate wear-out, while the pre-stressed ones will gradually relax. As depicted in Figure 6b, under regular operation, neither of the two monitors will go beyond a maximum degradation/relaxation window, i.e., the red box. In this scenario, both monitors will show that the IC has been operating trustfully;
- After nominal use and mild tamper annealing: in this situation, the chip has been used and tampered with to pretend that the IC is less used than it really is, like rolling back kilometers from an odometer in a car. In this scenario, the pre-stressed monitor enters its forbidden relaxation window because the anneal attempt accelerates its relaxation. When this happens, the tamper flag shows an illegitimate combination of the monitors, as depicted in Figure 6c, unveiling the tamper procedure and, more importantly, invalidating the IC age determination with the blue monitors;
- After a strong annealing procedure: in this scenario, the chip has undergone a strong annealing procedure to illegally rejuvenate it. In this case, both aging monitors will show almost complete relaxation status, as depicted in Figure 6d. Even though the IC seems to be brand new according to the first degradation monitor, the pre-stressed monitor is located deep in its forbidden region, revealing the strong annealing, maintaining the tamper awareness of the monitor system, and invalidating, also in this case, the IC’s age determination with the blue monitors.

Nevertheless, using regular-RO-based monitors reveals similar degradation levels for both static and dynamic operation, which indicates that BTI is also dominant in the latter [10]. To tackle this and enhance the orthogonality between the degradation kinetics of both monitors, which is a fundamental aspect of our monitoring concept, in this work, we have improved our HCD-driven monitor with a new layout design (True-HCD), and we have enhanced our regular-RO monitors to better exploit BTI degradation.

## 4. Results

This section will cover the extensive data obtained from the reliability tests conducted using our latest RO-based degradation monitor technology. It will describe the accelerated aging and annealing experiments conducted on our regular-RO-based monitors, as well as the novel True-HCD degradation monitor. Finally, this section will target a comparison of the annealing behavior of regular and True-HCD monitors to display the difference between the two monitors in terms of aging and annealing behaviors.

### 4.1. Evaluation Methodology

**Figure 7:**
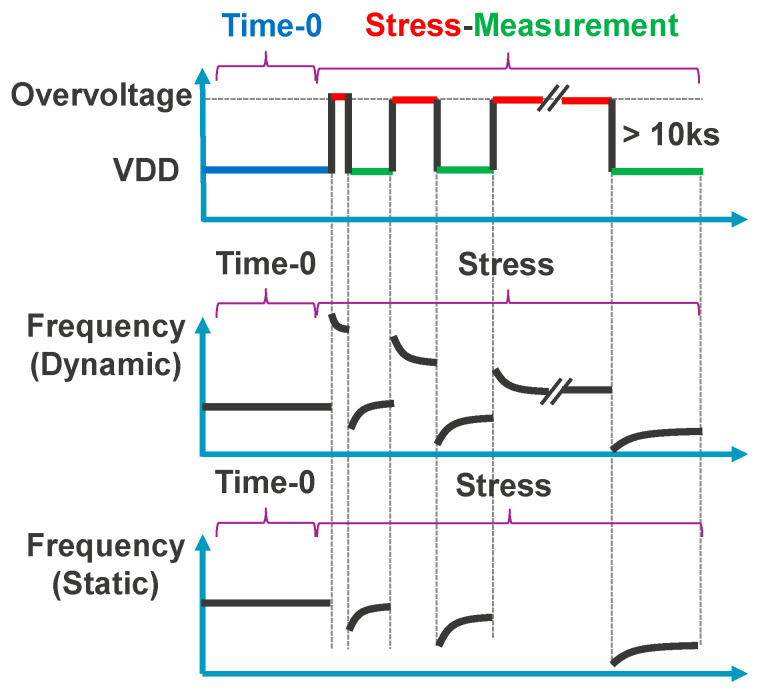
RO-based monitor eMSM test sequence: time-zero followed by eMSM tests: (**top**) VDD waveform, (**middle**) RO monitor frequency in dynamic stress and recovery, and (**bottom**) during static stress, where no frequency can be measured during stress phases.

All our degradation and annealing monitors utilized in this study have been subjected to enhanced Measurement Stress Measurement (eMSM) accelerated aging tests, as depicted in Figure 7 (top) [45,62,63,64]. The eMSM sequence consists of a set of concatenated stresses (Figure 7 red phases) and measurements (Figure 7 green phases), preceded by a fresh frequency readout, i.e., the non-stressed reference point (Figure 7 blue measurement phase). Nevertheless, depending on the type of reliability process to be investigated—degradation or annealing—different test procedures will be followed: for the degradation tests, eMSM is performed by means of overstress voltages, while for the annealing tests, a sequence analogous to the one used for the eMSM test is followed, but the stress phases are replaced by annealing phases in which the devices are unbiased and set at a high temperature, e.g., 325 °C.

On the one hand, when studying the accelerated degradation behavior of our monitors, during the stress phase, the bias of the RO is raised above the nominal technology voltage of 0.9 V, e.g., to 2.4 V, and once the stress time is finished, the monitor bias is switched back to the nominal supply voltage (in less than 100 µs) for frequency measurement after overvoltage stress. Moreover, in our eMSM test sequence, stress times are increased exponentially while measurement times are kept constant. In this study, we start with a stress time of 60 ms and increase it exponentially until 1000 s of accumulated stress. Each measurement period, also known as recovery, is kept fixed at 100 s. Since our degradation monitors are RO-based circuits, during the accelerated aging tests, we will distinguish two different types of stress modes: (i) when the RO feedback loop is closed (Figure 7, middle panel), it means dynamic stress and that the building transistors of the monitor undergo AC BTI and HCI degradation; and (ii) when the built-in RO feedback loop is open (Figure 7, bottom panel), it means static stress and that the RO is not oscillating, so constituent RO devices suffer DC BTI, while the building transistors in our new True-HCD mainly undergo DC HCD degradation.

On the other hand, when investigating the annealing behavior of the pre-stressed monitors, both the regular-RO-based and the true-RO monitor types are subjected to accelerated annealing by switching the annealing temperature between 25 °C and a high target temperature, e.g., 325 °C, as depicted in Figure 4 during a seven-cycle logarithmic annealing.

**Figure 8:**
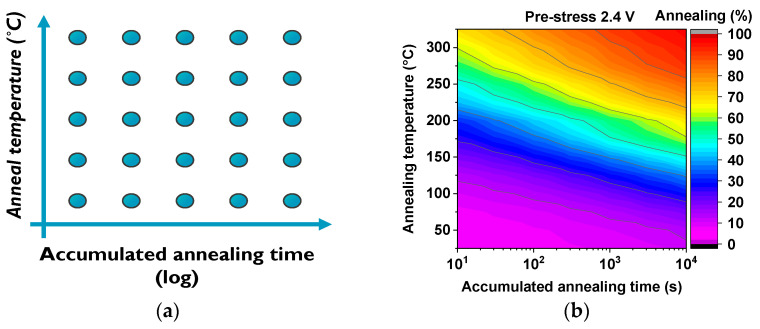
(**a**) Constellation of annealing experiments that are conducted to accurately obtain circuit annealing maps for temperatures starting from 25 °C up to 325 °C for accumulated annealing times as low as 10 s. (**b**) The resulting annealing map obtained by means of 13 pre-stressed ring oscillator circuits at 2.4 V during MSM of 3000 s and annealed for a total time of 10.000 s.

Like the stress period executed during the stress phases in eMSM testing, the high-temperature annealing cycles are increased exponentially in time to investigate how the annealing behaves under different temperatures and accumulated times, as exemplified in Figure 8a. During high-temperature annealing (Figure 4 red periods), the IC array is kept unbiased except for the heater, so the monitors do not receive any biasing to prevent unwanted monitor degradation during annealing. Then, immediately after the high-temperature phase has ceased and set back to 25 °C, the chip is biased, and the monitor is measured to evaluate the impact of the previous anneal cycle, which will depend on the applied temperature and the time that the monitor has been subjected to. As shown in the annealing map of Figure 8b, the dynamically pre-degraded frequency of each regular-RO monitor can be annealed, and the annealed percentage, shown from 25 °C to 325 °C with steps of 25 °C, reveals an initial resilience to annealing but at high temperatures and long annealing times, the frequency of the monitors can be almost completely annealed.

With the test methodologies described for accelerated stress temperature annealing by means of our built-in heaters, we will investigate and compare in the following subsections the behavior of regular-RO and then True-RO reliability monitors. Moreover, we will focus on how the new True-HCD monitor can be successfully used for IC age tracking because of its exceptional resilience to annealing compared with regular-RO-based monitors.

### 4.2. Regular-RO-Based Monitors: Frequency Degradation and Recovery

**Figure 9:**
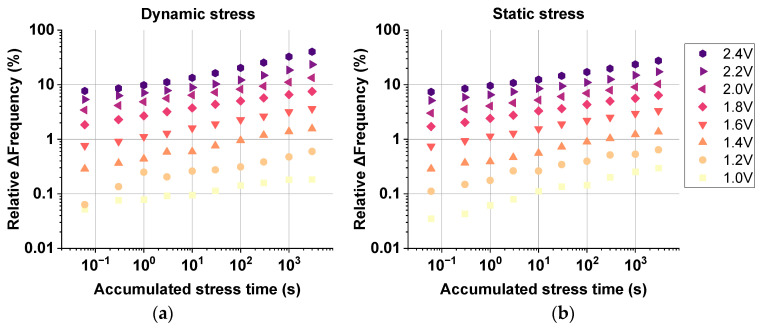
Degradation data exhibiting the power law dependence of regular-RO-based monitors. ∆Freq. (%) as a function of the accumulated stress time after (**a**) dynamic and (**b**) static stress for voltages ranging from 1.0 V to 2.4 V. The improved RO-array chip permits accurate characterization of ∆Freq. as low as 0.03% and allows important regular RO monitor degradation metrics such as the time exponent (*n*) and the voltage acceleration factor (VAF) to be easily obtained [33].

Our new IC design is the first to allow for the precise measurement of the degradation undergone by RO monitors both under dynamic and static operation at stress voltages close to the nominal technology voltage of 0.9 V. In this regard, Figure 9 shows the relative ∆Frequency (%) as a function of the accumulated stress time of regular-RO-based monitors stressed in dynamic mode, in Figure 9a, and in static mode, in Figure 9b, for voltages ranging from 1.0 V to 2.4 V. As clearly shown in both Figure 5a,b, the accumulated degradation is as low as ∆Freq. (%) ≈ 0.03% for a stress voltage of 1.0 V and stress time of 60 ms. Furthermore, the degradation data for all stress conditions follow a power law that permits the refinement of our RO-based degradation model in [30] and studies in [65,66], by accurately obtaining the n and VAF metrics at significantly low stress conditions, closer to the real chip operation.

It is important to emphasize that every symbol in Figure 9a,b corresponds to a set of two non-pre-stressed regular RO monitors that were aged by means of 10 consecutive stress and measurement cycles. Moreover, all measurement phases were executed for a total of 100 s each, with a brief time gap of 100 µs between removing the stress conditions and biasing the monitor with the nominal voltage of 0.9 V for recovery. The importance of accurately characterizing the recovery phase after each stress phase relies on the fact that charged traps in the building transistors of the monitor are released at different times after being charged during stress. Considering all the different stress conditions conducted, the dependence of RO frequency degradation on the accumulated stress time and the stress voltage, the time exponent (*n*), and the voltage acceleration factor (VAF) can be obtained from the data following a power law dependence in both cases. These dependencies can be expressed through the time exponent (*n*) and the voltage acceleration factor (VAF) for a power law degradation model (Δfreq = A·$t_{s}^{n}$·VDD^VAF^), as demonstrated in [31].

**Figure 10:**
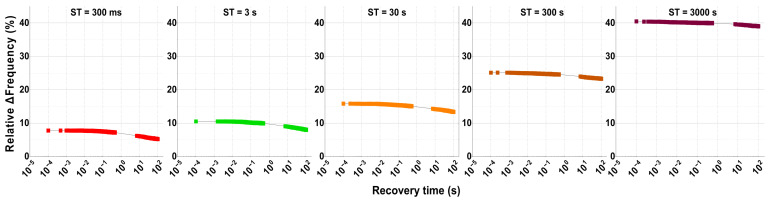
Display of the 5 recovery cycles measured after 10 stress cycles executed at 2.4 V on a regular-RO-based aging monitor stressed in dynamic mode.

To understand how the recovery phase affects and modulates the degradation data shown in Figure 9, Figure 10 displays all measured data collected during each recovery cycle in the 10-eMSM stress recovery test shown in Figure 9 for the highest stress voltage of 2.4 V in dynamic stress mode. Figure 10 depicts the 100 s recovery data, starting at the top left panel after the initial stress time of 60 ms and ending at the top right panel where the accumulated stress time of 3000 s has ceased. A clear non-linear behavior of the ∆Frequency (%) is observed in all panels, evidencing that after a few milliseconds, there is frequency recovery due to traps de-trapping during each recovery phase [54,65,67].

The recovery traces shown in all panels in Figure 10 first show that the ∆Frequency (%) starts at an elevated percentage value due to the previous stress. For instance, after only 60 ms of stress at 2.4 V, the ∆Frequency starts at ~5%, which can be considered a large degradation for such a small stress phase when compared to other works, like [68]. Moreover, if we focus on the last panel on the right, after an accumulated stress time of 3000 s and nine cycles of recovery, the ∆Frequency starts at ~40%. This also clarifies that, even after executing 100 s of circuit recovery at nominal voltage and room temperature after each stress phase, the accumulated degradation cannot be fully recovered, and more time and/or a higher temperature need to be applied to effectively de-trap the charges that were charged during the stress phases. Secondly, in all recovery phases, a plateau can be distinguished during the first microseconds, and, finally, after a few milliseconds into the recovery, the ∆Frequency (%) decreases, but nearly at the end of the recovery time window, it starts saturating. This behavior resembles universal BTI recovery [69,70].

**Figure 11:**
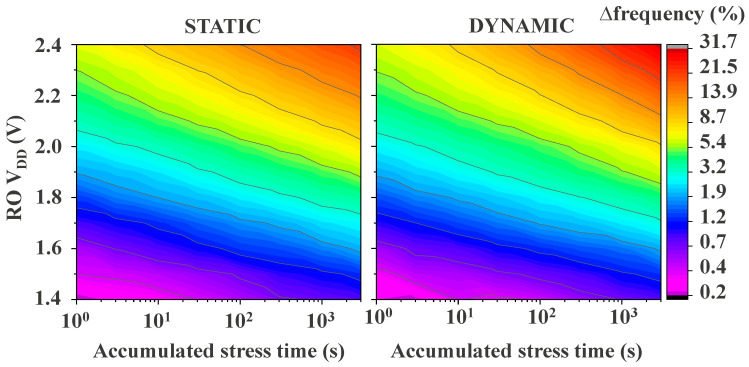
Static and dynamic degradation maps for overstress voltages ranging from 1.4 V to 2.4 V and stress times up to 3000 s. The maps are compiled utilizing the very first recovery point captured 100 µs after removing the stress conditions. The maps show the BTI degradation dominance over HCD except for high stress voltages and long stress times (purple triangles), highlighting the need to enhance HCD damage in dynamic RO stress.

This recovery behavior that is exhibited by all dynamically pre-stressed regular-RO-monitors in all recovery phases clearly determines that the predominant degradation phenomenon is mostly BTI. A clear example of BTI predominance is shown in Figure 11, where statically and dynamically degraded regular RO-based monitors reveal similar degradation levels in the bulk of both maps, except for the degradation reached at very high voltage and long stress times, e.g., RO VDD ≥ 2.3 V and *t*_stress_ > 1000 s. In addition, in the center of the maps, less degradation can be observed for dynamic stress than for static stress. Although the BTI component is predominant in both cases, with a mild contribution of HCD in the dynamic case, in static degradation, the induced BTI stress is DC, while in the dynamic mode, the BTI stress is AC; thus, BTI has less of an impact in the dynamic case. Nevertheless, as soon as the stress voltage and time are high enough, i.e., RO VDD ≥ 2.1 V and *t*_stress_ > 100 s, HCD starts to show up together with the BTI component, as seen in the top right corner, where there is ~10% more degradation in the dynamic than in the static mode.

BTI predominance clearly evidences the need to increase HCD degradation in RO-based monitors to fully exploit the distinct degradation kinetics of BTI and HCI degradation in circuits for IC age determination and accurate tamper detection [30,33]. Moreover, in in-field applications, available voltages are only CORE and IO, whereas internal Power Delivery Networks (PDNs) are only meant to provide stable CORE voltages to the chip. In this scenario, regular-RO-based monitors have proven to not be good candidates since, in in-field applications, higher-than-nominal voltages cannot be delivered and meaningful HCD degradation will be almost impossible to achieve.

### 4.3. True-RO-Based Monitors: Frequency Degradation and Recovery

**Figure 12:**
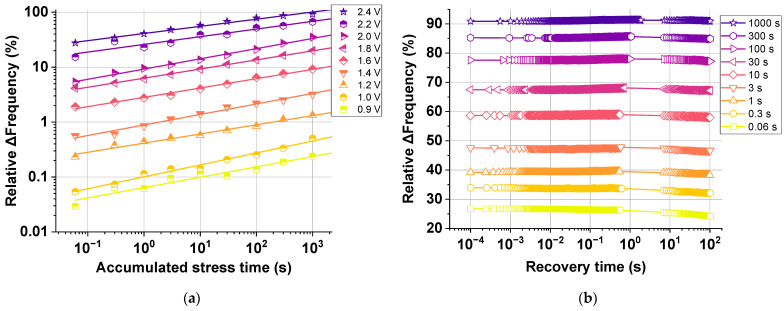
The new design was tested under different stress conditions with voltages ranging from 0.9 V up to 2.4 V, revealing power law degradation at the nominal voltage and ∆Freq. (%) > 90% when stressing at 2.4 V. (**b**) True-HCD monitor recovery traces after 9 stress cycles at 2.4 V and annealing map. In (**a**), the full recovery data set is shown, revealing almost no recovery, and reaching over ∆Freq. (%) > 90% after only 1000 s of stress.

To effectively achieve significant HCD degradation, this section will review our True-HCD monitor described in Section 2.2. This degradation monitor permits the individual stress of all pMOS or all nMOS building transistors of the built-in RO from the nominal technology voltage, i.e., 0.9, up to 2.4 V. To understand how the True-HCD monitor degrades and recovers, Figure 12a compiles the ∆frequency (%) of several True-HCD monitors degraded under a full range of stress voltages, i.e., from 0.9 V to 2.4 V. Figure 12a reveals meaningful degradation achieved when stressing in DC HCD even at the 0.9 V nominal technology voltage where, after 3000 s of accumulated stress time, a noteworthy ~0.2% of significant frequency degradation was achieved. Moreover, it is worth noticing that, when stressing at the highest stress voltage of 2.4 V for the same stress time of 3000 s, a significantly large ∆frequency of ~95% is achieved, mainly due to the large DC HCD degradation. If we compare the latter achieved degradation of the True-HCD monitor with the largest accumulated degradation of a regular-RO-based monitor, as shown in Figure 9a, the regular-RO could only reach ~40% of accumulated degradation due to the low effect of HCD degradation. Finally, as also shown in Figure 12a, the relative ∆Freq follows a power law, as also seen in the regular-RO data of Figure 9a,b.

The significant degradation achieved with the True-HCD monitor confirms that its design allows for the detection of degradation in in-field applications even if the chip has been operated at nominal voltage for a very short time, which would otherwise be undetectable by regular-RO monitors where all accumulated degradation will mainly be BTI.

To study the HCD degradation impact suffered by the True-HCD monitor, Figure 12b illustrates with dark green stars the complete nine-cycle recovery data of the True-HCD monitor stressed at 2.4 V in Figure 12a. In this case, every symbol of the 2.4 V-stressed True-RO monitor in Figure 12a corresponds to the very first data point, i.e., at 100 µs of recovery time, in Figure 12b. The recovery data shown clearly exhibit nearly flat recovery traces during the entire seven decades of recovery traces. This behavior reinforces the conclusion that the lack of frequency recovery is a direct consequence of mainly having HCD degradation in the building transistors of the True-HCD monitor with an almost negligible recoverable component [44]. This is of extreme importance because HCD degradation is more resilient to recovery when compared with BTI degradation.

### 4.4. Tamper-Flag Capability of the True-HCD Monitor

The tamper-flag capability of our on-chip degradation monitor technology consists in taking advantage of the relaxation process of the highly pre-stressed True-HCD monitors (Figure 6a, red oval). In this context, understanding the annealing process of pre-stressed monitors at different voltages, annealing times, and temperatures is crucial. In this scenario, this subsection will show extensive and accurate on-chip annealing experiments conducted on highly pre-stressed regular-RO and True-HCD monitors.

We show how regular-RO and true-HCD degradation monitors anneal after being stressed from low to high stress voltages. For this purpose, an extensive set of pre-stress and annealing tests was conducted on a total of 286 fresh monitors, 130 regular-RO monitors, and 156 true-HCD monitors.

**Figure 13:**
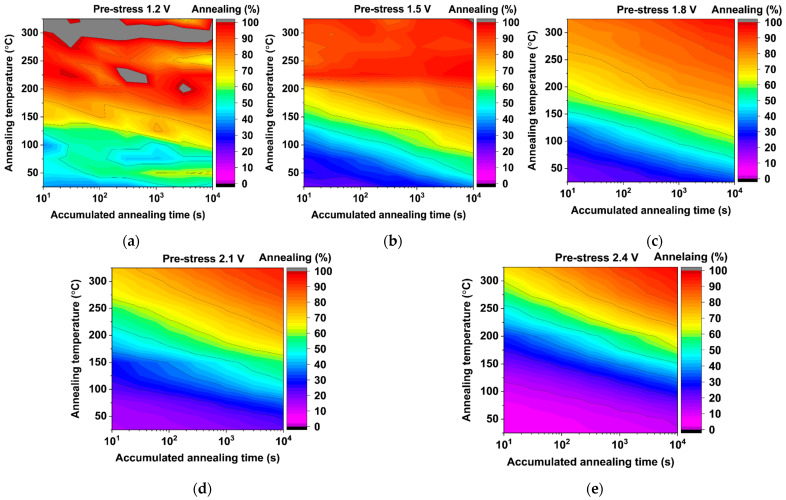
On-chip temperature annealing maps were conducted from 25 °C to 325 °C on dynamically pre-stressed regular-RO-based monitors at (**a**) 1.2 V, (**b**) 1.5 V, (**c**) 1.8 V, (**d**) 2.1 V, and (**e**) 2.4 V. All maps reveal a color progression from blue (mild anneal) to red (strong anneal) for low pre-stress voltages, (**a**–**c**), while the highly pre-stressed regular-RO monitors (**d**,**e**) anneal from pink (low anneal) to red. Maps (**d**,**e**) expose some resilience to annealing, but the red zone present in both of them emphasizes the fact that all pre-stressed regular-RO-based monitors can be almost completely annealed.

**Figure 14:**
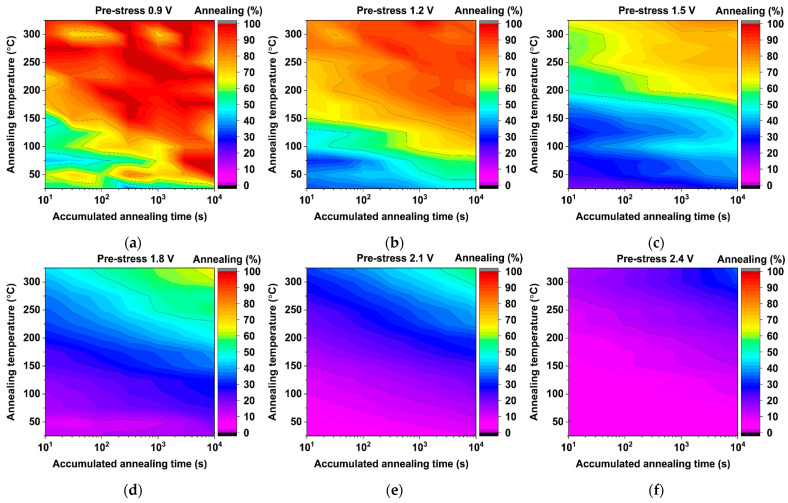
On-chip temperature annealing maps conducted on pre-stressed True-HCD monitors at (**a**) 0.9 V, (**b**) 1.2 V, (**c**) 1.5 V, (**d**) 1.8 V, (**e**) 2.1 V, and (**f**) 2.4 V. Like in Figure 13a, an almost complete anneal can be achieved in (**a**,**b**) due to the low HCD degradation. Nevertheless, the achievable annealing of pre-stressed monitors as from 1.5 V becomes narrower with increasing stress voltages due to the DC HCD large degradation reaching only a maximum of ~25% when pre-stressing the true-HCD monitors at 2.4 V.

All annealing maps shown in Figure 13 and Figure 14 harbor 13 annealing temperature conditions, i.e., from 25 °C to 325 °C in steps of 25 °C, where each annealing test was conducted two times using different monitors. All annealed devices were pre-stressed using the accelerated eMSM aging sequence described in Section 4.1, reaching 3000 s of accumulated stress time. After that, all monitors were annealed for up to 10,000 s of accumulated annealing time, i.e., 10 s, 30 s, 100 s, 300 s, 1000 s, 3000 s, and 10,000 s. In summary, each monitor was tested for a total of 14,000 s, consisting of eMSM stress for 4,000 s and an annealing procedure for 10,000 s, which amounts to approximately 4 h. Then, to complete the 11 annealing maps shown in Figure 13 and Figure 14, a total of ~46 days of testing were required to test the 286 monitors.

On the one hand, annealing results shown in Figure 13a, starting with low pre-stress voltages of 1.2 V, show light blue and green annealing regions, proving easily degraded monitor annealing, i.e., >45%, achieved at 25 °C. This is a consequence of the low pre-stress voltages applied to the regular monitors, where degradation is so low that almost 50% can be annealed at room temperature. Moreover, for higher annealing temperatures, i.e., >150 °C, the degradation can be completely annealed out, reaching ≥100%. On the contrary, as soon as the pre-stress voltage increases from 1.5 V to 2.4 V, i.e., Figure 13b–e, the achievable annealing of the regular monitors decreases because of the larger degradation achieved in the pre-stress phase. Moreover, in Figure 13b,c, a dark blue region appears, revealing that lower annealing percentages can be achieved at low temperatures, i.e., ~25%. Finally, looking at annealing maps (d) and (e) in Figure 13, a clear pink region emerges at 25 °C, indicating that the monitor shows much more resilience to annealing at low temperatures due to the damage suffered during the previous dynamic degradation. Nevertheless, it is important to note that all annealing maps shown in Figure 13 harbor a dark red region, proving that over ~95% of accelerated degradation can be effectively annealed by applying high temperatures and sufficient annealing times.

On the other hand, the new True-HCD monitors prove to be much more resilient to annealing, as can be observed by the lack of red regions for pre-stress voltages starting from 1.5 V, Figure 14c–f. In this case, the higher the stress voltage, the lower the achievable annealing. For instance, in the extreme case of 2.4 V of pre-stress, the True-HCD monitors can be annealed up to a maximum of ~30%, while a regular-RO-based monitor has proven to achieve more than 95%, as shown in Figure 13e.

When annealing the pre-stressed True-HCD monitors, the achievable anneal, as shown in Figure 14a, reaches up to 100%. This is a consequence of the very low HCD degradation accumulated by the monitor during the pre-stress phase. Furthermore, as soon as the stress voltage increases, the annealing percentage starts rapidly decreasing. This behavior exposes more HCD degradation, characterized by charged traps with very long emission times, as well as interface states [54]. This hinders recovery through high-temperature annealing, revealing no significant frequency anneal even in a long anneal window of 10,000 s. In summary, it has been proven that the True-HCD monitor is mainly degraded by DC HCD aging and is much more resilient to high-temperature annealing due to DC HCD degradation when compared with regular BTI-dominated monitors.

**Figure 15:**
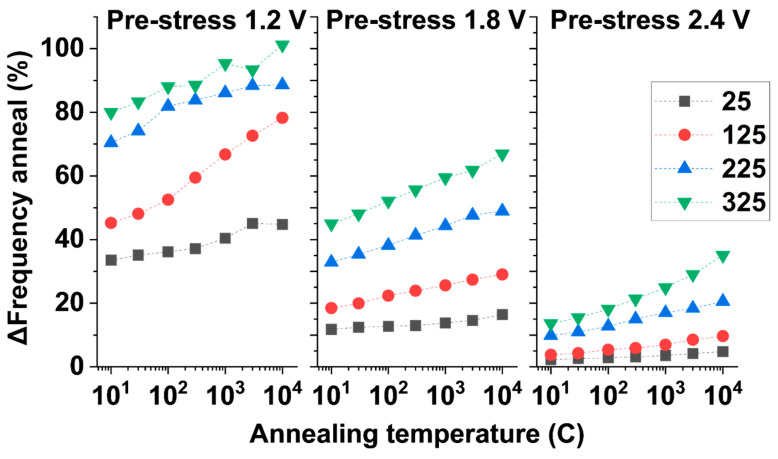
Comparison graph of ∆frequency annealing for 3 pre-stress voltages, 1.2 V, 1.8 V, and 2.4 V, and at 4 different annealing temperatures.

An in-depth comparison of the achievable annealing of our True-HCD aging degradation monitor is shown in Figure 15. The figure compares the percentage of annealed frequency for low (1.2 V), mid (1.8 V), and high (2.4 V) pre-stressed monitors and how the annealing at four different temperatures recovers the pre-degraded frequency of the involved monitors, as also shown in the degradation maps in Figure 14. We can observe that the lower the pre-stress voltage, e.g., 1.2 V, and the higher the annealing temperature, for instance, 325 °C, the fuller the annealing of the monitor, as shown with green triangles in Figure 15 after 10,000 s of accumulated annealing at 325 °C a pre-stress of 1.2 V. Moreover, as soon as the pre-stress voltage is increased, from 1.8 V to 2.4 V, the maximum annealed frequency drastically decreases to ~65% and ~30%, respectively, due to the large DC HCD degradation experienced by the monitor in the pre-stress phase. The frequency recovery achievable through high-temperature annealing shows that the traces are almost parallel, and smaller distances appear with increasing pre-stress voltages. For instance, when pre-stressing the True-HCD monitors at 2.4 V, the annealing percentages for short annealing times are very close together, and only for longer annealing times do the annealed frequency traces diverge. All this behavior combined determines how the True-HCD monitors anneal as a function of time and temperature after being pre-stressed. During in-field operation, the use of pre-stressed True-HCD monitors would be extremely useful because the monitors would anneal during the lifetime of the chip while being unbiased and sensitive to temperature variations, with different ratios depending on the pre-stress conditions. In this scenario, the pre-stressed True-HCD monitors will remain disconnected from VDD and only momentarily biased to measure its status when required. On the contrary, non-pre-stressed True-HCD monitors will accumulate degradation during in-field operation for lifetime assessment and predictive maintenance purposes. In summary, the combination of both monitors on-chip, i.e., pre-stressed and non-pre-stressed, during in-field operation will increase the accuracy of the IC’s lifetime extraction converting the proposed on-chip degradation monitor technology into a unique source of chip security.

### 4.5. Utilizing Annealing of True-HCD Monitors for IC Age Determination

The main usage of the True-HCD degradation monitor for in-field applications would be as a tamper flag to detect high-temperature annealing events, as described in Figure 6. This annealing event could occur because of fraudulent attempts to roll back the accumulated degradation by the degradation monitors by means of high-temperature application or because of high temperature applied during soldering and desoldering procedures. In both cases, the pre-stressed True-HCD monitors will also be present in the IC and, as depicted in Figure 6, will be able to track IC annealing during regular- or high-temperature events that may happen during in-field operation. Moreover, the True-HCD monitor’s annealing behavior could also be used to complement the aging degradation data utilized to obtain the remaining lifetime of the chip with its annealing data.

**Figure 16:**
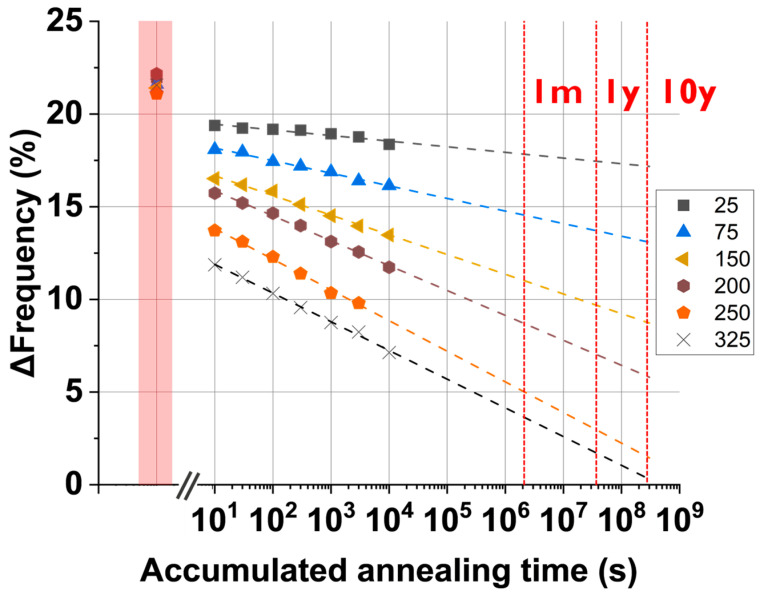
Annealing profiles of True-HCD monitors were obtained for annealing temperatures from 25 °C to 325 °C. The graph shows the measured annealing data up to 10,000 s and linear fitting extrapolations up to 1 month (1 m), 1 year (1 y), and 10 years (10 y) with red-dashed vertical lines.

As shown in the previous section, the True-HCD monitor has been proven to anneal at different ratios depending on the pre-stress voltage, the annealing temperature, and the annealing time. Understanding how a pre-stressed True-HCD monitor anneals in a long-term time window can be utilized to determine the age of the chip. In this scenario, Figure 16 shows how seven True-HCD monitors pre-stressed at 1.8 V, i.e., the I/O technology voltage, anneal at different annealing temperatures. In the figure, the ∆Frequency (%) is shown as a function of the accumulated annealing time, except for the red square section, which shows the degradation level reached for each monitor after being pre-stressed, i.e., ~21%. Then, annealing data from measurements executed in a time window that extends up to 10,000 s are shown. We also extrapolated the fittings until an annealing time of 10 years. It is worth pointing out that for higher annealing temperatures, we encounter a higher slope, which means that more pre-stress degradation will be recovered.

The annealing behavior shown in Figure 16 is of extreme importance when dealing with annealing monitors because regular-RO monitors, where BTI is predominant, might not achieve the accuracy and resilience needed for long-term utilization, e.g., 10 years. In this sense, the annealing frequency profiles of the True-HCD monitors show varying annealing slopes depending on the IC temperature during the annealing.

For instance, if the IC is operated at 75 °C, it has a less steep slope than if compared with an IC that is operated at 150 °C, i.e., automotive-grade temperature. In summary, with the data shown in Figure 16, the age of the IC can be determined by the percentage of annealing frequency together with our degradation model of all True-HCD monitors involved, which will depend on the IC temperature profile during in-field operation.

**Figure 17:**
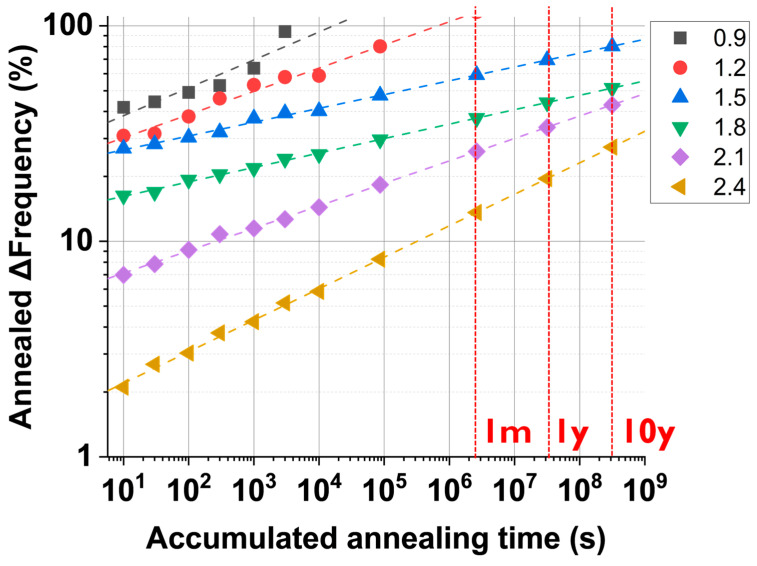
Annealed ∆Frequency (%) as a function of the accumulated annealing time for several True-HCD monitors pre-stressed with voltages from 0.9 V until 2.4 V annealed at 75 °C. The graph shows the gathered data from our IC arrays up to 10^4^ s, as well as the extrapolated linear fitting and data points up to 1 month (1 m), 1 year (1 y), and 10 years (10 y) defined in the graph with red-dashed vertical lines.

Another inflection variable that must be taken into consideration when utilizing True-HCD monitors is the pre-stress voltage applied during the wafer sort phase. Depending on the pre-stress voltage, the annealing kinetics will differ and can be used to cross data for the purpose of accuracy when extracting the age of a chip. In this scenario, Figure 17 shows the annealed ∆frequency of pre-stressed True-HCD monitors at voltages from 0.9 V up to 2.4 V with steps of 0.3 V. Each pre-stressed monitor was subjected to the same annealing sequence at 75 °C, as described in Figure 8a. The data were linearly fitted and extrapolated up to 10 years (10 y) but keeping the maximum monitor annealing at 100%. As depicted in Figure 17 in different colors, each pre-stress voltage utilized exhibits a different slope that defines how that pre-stressed monitor will anneal in time, up to 10,000 s in the lab but also in 10 years.

The annealing behavior of our True-HCD degradation monitor during circuit annealing, as gathered in Figure 16 and Figure 17, is key to defining and modeling the behavior of the monitor in terms of pre-stress voltage, IC temperature, and IC operation time. During in-field operation, the monitor is measured upon request for a few microseconds to determine the annealing status and project the age of the chip, as well as the remaining lifetime depending on the maximum degradation target of each chip.

## 5. Conclusions

In this work, we have presented our most recent on-chip degradation monitor technology, which also includes a novel tamper-aware concept capable of identifying fraudulent high-temperature annealing. This technology utilizes two sets of ring oscillator-based aging monitors, one to account for IC degradation and another set of highly pre-stressed monitors utilized to detect fraudulent high-temperature IC annealing. To this end, we reviewed and utilized our latest IC array, designed and fabricated using a 28 nm commercial technology, that harbors hundreds of on-chip degradation monitors.

Our novel IC sub-array comprises a total of 112 addressable unit cells that harbor ring oscillator-based degradation monitors covering regular-RO designs and our novel True-HCD degradation monitor. The array incorporates a dedicated circuit design that ensures the ability to perform trustworthy characterization of our degradation monitors in terms of accelerated reliability testing. The addition of an on-chip Force-and-Sense voltage biasing system ensures that, during variability characterization, on-chip voltage drop is mitigated, and all defined voltages are correctly applied to the test devices. The IC I/O transmission gates allow for the application of stress voltages to the monitors up to 2.4 V and for sustaining up to 3 mA of DC current. Moreover, the chip also incorporates a novel on-chip heater structure designed in the FEOL with N+ diffusion resistors that allows on-chip homogeneous and fast temperature setting of ∆T = +300 °C.

A large set of RO-based monitors, between regular-RO and True-HCD monitors, was tested, and the degradation and annealing data collected during an extensive set of reliability tests were presented and analyzed, revealing the degradation and recovery behaviors of the aging and annealing monitors. More specifically, True-HCD pre-degraded monitor annealing data proved that the monitors are much more resilient to high-temperature annealing compared to regular-RO-based aging monitors, even when stressed in a dynamic mode and dominated by BTI. Finally, we also show that True-HCD monitors that were intentionally pre-stressed proved to be the perfect candidate to unequivocally detect fraudulent high-temperature IC anneals and execute the predictive maintenance of ICs by combining degradation and annealing data coming from our monitors in in-field applications.

## Figures and Tables

**Table 1:** Biasing conditions of the True-HCD aging monitor.

Target Devices	RO VDD = StressV V_INT_ = 0 V	RO VDD = V_INT_ = StressV
pMOS	V_DS_ = V_GS_ = −StressV	V_DS_ = V_GS_ = 0 V
nMOS	V_DS_ = V_GS_ = 0 V	V_DS_ = V_GS_ = StressV

## Data Availability

Data can be provided upon request.

## Abbreviations

The following abbreviations are used in this manuscript:

BTI	bias temperature instability
CPU	Central Processing Unit
eMSM	Enhanced Measurement Stress Measurement
F&S	Force-and-Sense
HPC	high-performance computing
HCD	hot carrier degradation
IC	integrated circuit
IO	Input/Output
*n*	Time acceleration factor
PDN	Power Delivery Network
RO	ring oscillator
V_INT_	Internal Voltage
Vj	junction voltage
VAF	voltage acceleration factor
